# Skeletal Muscle Abnormalities in Pulmonary Arterial Hypertension

**DOI:** 10.1371/journal.pone.0114101

**Published:** 2014-12-02

**Authors:** Ana Paula Breda, Andre Luis Pereira de Albuquerque, Carlos Jardim, Luciana Kato Morinaga, Milena Mako Suesada, Caio Julio Cesar Fernandes, Bruno Dias, Rafael Burgomeister Lourenço, Joao Marcos Salge, Rogerio Souza

**Affiliations:** 1 Pulmonary Department, Heart Institute University of São Paulo Medical School, Sao Paulo, Brazil; 2 Radiology Department, University of São Paulo Medical School, Sao Paulo, Brazil; Indiana University, United States of America

## Abstract

**Background:**

Pulmonary arterial hypertension is a progressive disease that is characterized by dyspnea and exercise intolerance. Impairment in skeletal muscle has recently been described in PAH, although the degree to which this impairment is solely determined by the hemodynamic profile remains uncertain. The aim of this study was to verify the association of structural and functional skeletal muscle characteristics with maximum exercise in PAH.

**Methods:**

The exercise capacity, body composition, CT area of limb muscle, quality of life, quadriceps biopsy and hemodynamics of 16 PAH patients were compared with those of 10 controls.

**Results:**

PAH patients had a significantly poorer quality of life, reduced percentage of lean body mass, reduced respiratory muscle strength, reduced resistance and strength of quadriceps and increased functional limitation at 6MWT and CPET. VO_2_ max was correlated with muscular variables and cardiac output. Bivariate linear regression models showed that the association between muscular structural and functional variables remained significant even after correcting for cardiac output.

**Conclusion:**

Our study showed the coexistence of ventilatory and quadriceps weakness in face of exercise intolerance in the same group of PAH patients. More interestingly, it is the first time that the independent association between muscular pattern and maximum exercise capacity is evidenced in PAH, independently of cardiac index highlighting the importance of considering rehabilitation in the treatment strategy for PAH.

## Introduction

Pulmonary arterial hypertension (PAH) is a severe disease characterized by progressive remodeling of small arteries, which results in elevated pulmonary vascular resistance and right ventricular failure [1]. Despite recent advances in therapeutic alternatives [2], exercise tolerance and even survival in PAH remains poor [3]–[6]. Dyspnea on exertion and exercise limitation are highly prevalent features of PAH and represent the most disabling factors in these patients, independent of the presence of associated conditions [7]. Although hemodynamic impairment is considered to primarily be responsible for this exercise intolerance, cardiac output only partially correlates with exercise capacity, which suggests the existence of other limiting factors [8]. The involvement of ventilatory muscles was first described independent of pulmonary hemodynamics [9]. Subsequently, the same group observed not only ventilatory weakness but also reduced forearm strength in PAH patients [10]. In parallel with similar chronic diseases (COPD and left heart failure), recent studies suggest that PAH patients may also present peripheral muscle dysfunction in the lower extremities [8]; furthermore, rehabilitation programs might be beneficial for these patients [11], [12]. Nevertheless, the issue of whether these skeletal muscle abnormalities are unique consequences of the hemodynamic profile of PAH or whether they play an independent role in exercise limitation is still open to debate.

The aim of this study was to verify the association between structural and functional skeletal muscle characteristics and the maximum exercise capacity of PAH patients.

## Methods

### Subjects

Sixteen patients with a diagnosis of PAH, according to international guidelines, [13] were recruited. All patients had mPAP≥25 mmHg and wedge pressure <15 mmHg that was measured by right heart catheterization. Six patients had schistosomiasis-associated PAH (Sch-PAH) that was diagnosed according to previously described criteria [14], [15]. Clinical stability and no change in treatment during the previous 8 weeks were mandatory criteria for study enrollment. The exclusion criteria were: (1) left heart diseases with ventricular ejection fraction <55%; (2) pulmonary hypertension secondary to connective tissue diseases and HIV; (3) oxygen dependence; (4) diabetes mellitus; and (5) neuromuscular disorders. Ten healthy but not physically active individuals were recruited as a control group. The protocol was approved by the ethical committee, and informed consent was obtained from all individuals.

All tests were performed in three consecutive visits with a minimum interval of 24 hours.

### Day 1

The health related quality of life questionnaire Short Form-36 (SF-36) [16] was used for all patients and controls. The Physical Component Summary (PCS) and Mental Component Summary (MCS) were derived from the eight domains of the questionnaire. Total body composition was determined based on the impedance to electrical flow in different tissues (lean mass and fat), using the *Biodynamics 310 (Biodynamics Corporation, EUA)*.

Spirometry and lung volume measurements were performed using the EliteD body box (Medical Graphics Corporation - MGC, St Paul, Minn, USA) according to American Thoracic Society/European Respiratory Society (ATS/ERS) recommendations. Maximal voluntary ventilation (MVV, L) was determined indirectly as the product of FEV_1_ × 37.5 [17], [18]. The predicted values were obtained using *Brazilian* standards [19]–[21]. Maximal static inspiratory and expiratory mouth pressures (MIP and MEP, respectively) were measured using a portable mouth pressure meter (Gerar, São Paulo, Brazil). MIP was calculated from the residual volume level, and MEP was derived from total lung capacity, performed in a seated position, using the ATS/ERS guidelines [22].

A six-minute walk test (6MWT) was performed according the ATS/ERS recommendations [23]. Finally, patients performed an isokinetic knee-extensor test at 60°.s^−1^ to record the peak torque (N.m^−1^) and at 240°.s^−1^ for total work (J) in the *Biodex (Biodex Multi-Joint System3, Shirley, NY)*.

### Day 2

A computed tomography (CT) scan of the dominant thigh was performed with the individual lying down using the *Philips Medical Systems™ (iSite PACS 4.1 Philips Health Care Informatics; CA, USA)*. A 12 mm image taken at the halfway point between the femur and the lateral condyle was analyzed, and the muscle cross-sectional area was quantified based on the surface area of the muscle tissue (35–100 Hounsfield units).

A symptom-limited incremental exercise test was then performed on an electromagnetically braked device (Corival, Lode BV, Groningen, Netherlands) with subjects connected to a flow-meter mouthpiece. The subjects were asked to maintain a pedaling frequency of 60±5 min^−1^ in a protocol composed of the following: (i) 2 min at rest; (ii) 2 min of unloaded cycling; and (iii) an incremental phase (5 to 15 W.min^−1^) adjusted to provide a test duration of 8–12 min. Metabolic (oxygen uptake and CO_2_ output) and ventilatory (minute ventilation and respiratory rate) parameters were measured breath by breath, and the mean of the last 20 sec of every minute was calculated. Predicted values were those of the adult Brazilian population [24].

### Day 3

A biopsy of the vastus lateralis muscle was performed in the dominant leg at midthigh. Patients were instructed to stop taking any prescribed oral anticoagulation medication in the 7 days prior to the procedure, and the international normalized ratio (INR) was measured before the procedure. Local anesthesia was used (Xylocain 1%), and a skin incision of 2 cm was made by a trained physician. Four samples of 0.5 mm^3^ were obtained and frozen in Nitrogen liquid at −80°C. Transverse sections of 6–8 µm were cut.

The proportion of fiber types was measured based on immunohistochemical staining as follows: type I (myosin*-1, anti-mouse, clone A4.951, Hybridoma Bank*), type II (*myosin-2, anti-mouse, clone WB-MHCf, Novocastra*) and type IIa (*anti-mouse; clone SC-71; Hybridoma Bank*). The percentage of each type of muscle fibers was calculated from the total fiber number. The surface of 400 fibers was calculated manually by a trained technician.

### Statistical analysis

Continuous data are presented as mean+standard deviation (SD), while categorical data are presented as ratios. Comparisons between PAH and control groups were performed using unpaired T tests and Fisher’s exact tests, as appropriate. A Pearson’s correlation method was used to evaluate the association between variables of interest. Bivariate linear regression models were built to verify the association between muscular functional and structural variables with maximum VO_2_.

## Results

Demographic, functional and hemodynamic data are shown in table 1. The study group was composed of 10 patients (62.5%) with IPAH and 6 patients (37.5%) with schistosomiasis-associated PAH (Sch-PAH), while the control group was composed of 10 healthy subjects. No difference in gender and body mass index was found between the groups; however, the PAH patients were older than the control subjects mainly due to patients with Sch-PAH. No statistical difference was found when comparing spirometry data, although a trend towards a smaller forced vital capacity was observed. The maximal inspiratory and expiratory pressures, however, were significantly decreased in PAH patients. Finally, although the MCS was not different between groups, PAH patients had worse PCS according to the SF-36 questionnaire.

**Table 1:** General characteristics.

	PAH	Controls	p
Anthropometric data			
Age, yrs.	41.6 (13.3)	34.1 (3.1)	0.046*
Gender, male/female	4/12	3/7	0.780
Body Mass Index, Kg/m^2^	25.4 (3.4)	24.5 (4.2)	0.542
Lung Function			
FVC, % predicted	86.8 (17.6)	112.3 (43.6)	0.053
FEV_1_, % predicted	87.6 (19.6)	94.6 (16.4)	0.361
FEV_1_/FVC, % predicted	81.0 (6.3)	75.1 (16.3)	0.296
MIP, cmH_2_O	−61.9 (14.6)	−92.0 (17.3)	<0.001*
%predicted	64.2 (16.9)	89.3 (17.0)	0.001*
MEP, cmH_2_O	89.0 (20.6)	116.0 (20.4)	0.003*
%predicted	89.4 (18.5)	110.2 (25.2)	0.023*
SF-36 Health Survey			
Physical Component Summary	38.5 (12.3)	51.5 (5.0)	0.001*
Mental Component Summary	48.8 (12.8)	48.2 (8.1)	0.887
Functional Class - *NYHA*/WHO			
% II	68.8		
% III	31.3		
Pulmonary Hemodynamic			
RAP, mmHg	9.1 (4.0)		
mPAP, mmHg	60.0 (18.3)		
Wedge, mmHg	10.9 (3.0)		
CI, L/min/m2	2.69 (0.84)		
CO, L/min	4.7 (1.6)		

PAH; pulmonary arterial hypertension; FVC: forced vital capacity; FEV_1_: forced expiratory volume in 1^st^ second; MIP: maximal inspiratory pressure; MEP: maximal expiratory pressure; NYHA: New York Heart Association; RAP: right atrial pressure; mPAP: mean pulmonary arterial pressure; CO: cardiac output. Data are presented as Mean (SD), *p<0.05.

Two thirds of the PAH group presented with NYHA/WHO functional class II disease. Hemodynamic data were compatible with previously described PAH populations [14].

### Muscle function and morphology

PAH patients had lower lean mass as determined by bioelectrical impedance, but there was no decrease in thigh muscle area when measured by CT. Quadriceps muscle function had reduced force generation capacity during short repetitions (peak torque) and in the endurance test (total work) when compared with controls (Table 2).

**Table 2:** General characteristics of peripheral muscles.

	PAH	Controls	p
*Bioelectrical impedance*			
% fat mass	28.6 (3.6)	24.6 (4.4)	0.019*
% lean mass	71.4 (4.9)	76.2 (6.6)	0.044*
*Thigh CT*			
Area, cm^2^	108.5 (25.9)	120.5 (34.9)	0.323
* Strength*			
Peak Torque (60°/s)			
Absolute (N.m^−1^)	108.9 (31.9)	147.0 (39.3)	0.012*
Total work (240°/s)			
Absolute (J)	659.6 (242.8)	932.7 (295.6)	0.017*

Data are presented as Mean (SD), *p<0.05.

A biopsy was performed only in the PAH group. Although surface area was similar for different fibers, there was predominance in the percentage of type IIa and IIx fibers when compared with the surface area of type I fibers (Table 3). Comparing to previous data obtained from healthy persons with similar ages, our patients had lower proportion of type I and IIa fibers [25].

**Table 3:** Characteristics of muscle biopsies in vastus lateralis from PAH patients.

Muscle biopsy	PAH	Controls*
Fiber typing (%)		
% Type I	27.4 (8.6)	44 (16)
% Type IIa	33.7 (8.5)	46 (9)
% Type IIx	38.8 (8.5)	
Fiber type surface area (µm^2^)		
Type I	3326.7 (736.0)	
Type IIa	3061.8 (1222.7)	
Type IIx	3029.2 (868.7)	

*reference 25.

Data are presented as Mean (SD).

### Exercise capacity

The distance walked in the 6MWT was reduced in the PAH group when compared with the control group (490 m vs. 632 m, p<0.001) (Table 4). In the maximal cardiopulmonary exercise test, both groups performed a symptom-limited exercise test that is characterized by high respiratory exchange ratios and increased symptoms. PAH patients had decreased exercise performance that was confirmed by the workload and VO_2_ at test cessation. Although the PAH patients already had increased minute ventilation and respiratory rates at rest (data not shown), they showed an even greater increase in ventilatory response (Delta V_E_/VCO_2_) than the control group, despite their lower tidal volume at peak exercise. Their P_ET_CO_2_ decreased substantially during effort progression, in contrast to the increase in the physiologic response in control subjects. Finally, the PAH patients were not capable of achieving a maximal HR and showed a lower oxygen pulse at test cessation.

**Table 4:** Six-minute walk test and CPET data.

	PAH	Controls	P
*Peak 6MWT*			
Distance, m	490.8 (85.3)	632.1 (34.7)	<0.001
Distance, % predicted	87.5 (14.1)	109.5 (7.2)	<0.001
SpO_2_, %	92.1 (4.6)	96.9 (1.2)	0.001
HR, bpm	126.7 (14.8)	125.7 (18.2)	0.881
HR, % predicted	70.8 (7.6)	68.3 (10.0)	0.469
*Peak Incremental Test*			
Workload, W	82.9 (27.7)	129.2 (30.2)	0.001
Workload, %predicted	60.5 (18.6)	86.2 (12.3)	0.001
VO_2_, ml.Kg^−1^min^−1^	14.7 (3.5)	22.9 (2.8)	<0.001
VO_2_,%predicted	54.8 (17.0)	74.6 (6.4)	0.002*
RER	1.1 (0.1)	1.25 (0.1)	0.027
V_E_, L.min^−1^	53.5 (16.7)	61.3 (13.0)	0.218
Delta V_E_/VCO_2_	44.1 (6.5)	30.3 (2.1)	0.001
RR, rpm	37.0 (8.7)	34.6 (6.2)	0.445
Tidal volume, ml	1.46 (0.44)	1.78 (0.35)	0.068
P_ET_O_2_, mmHg	110.0 (8.5)	103.6 (5.7)	0.045
P_ET_CO_2_, mmHg	25.8 (6.9)	34.6 (4.7)	0.002
SpO_2_, %	94.2 (3.9)	97.0 (1.2)	0.038
HR, bpm	145.6 (15.8)	162.2 (16.3)	0.018
HR, % predicted	81.2 (8.1)	88.1 (8.7)	0.051
O_2_Pulse, ml.bpm^−1^	6.75 (2.0)	9.9 (3.8)	0.013*
O_2_Pulse, % predicted	65.9 (20.5)	84.8 (10.9)	0.014*
Borg dyspnea	4.9 (2.7)	4.6 (2.8)	0.813
Borg leg	5.7 (3.1)	6.1 (2.1)	0.708

PAH: pulmonary arterial hypertension; SpO_2_: oxygen pulse saturation; HR: heart rate; VO_2_: oxygen consumption expired; VCO_2_: carbon dioxide expired; P_ET_O_2_: partial pressure of oxygen at end expiration; P_ET_CO_2_: partial pressure of carbon dioxide at end expiration; RER: respiratory exchange ratio; V_E_: minute ventilation; RR: respiratory ratio; O_2_pulse: oxygen pulse. Data are presented as Mean (SD), *p<0.05.

**Figure 1:**
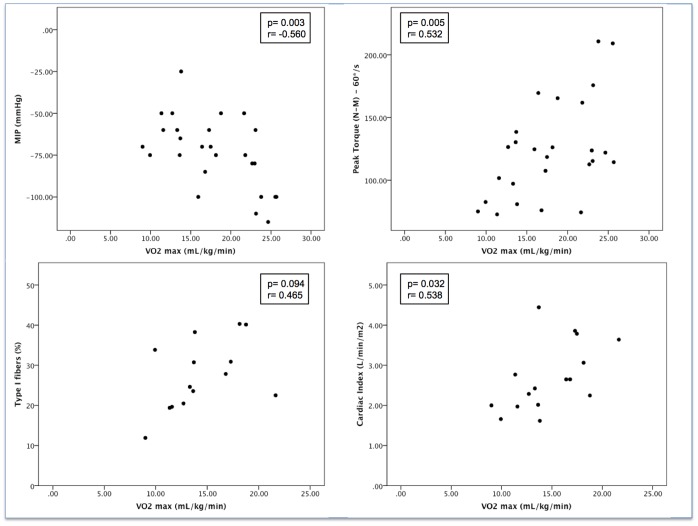
Correlation between VO_2_ max (ml/min) and A – MIP; B – Peak torque; C – Percentage of type I fibers; D – Cardiac Output.

The VO_2_max was negatively correlated with maximum inspiratory pressure and was positively correlated with peripheral strength and cardiac index (Figure 1– A, B and D, respectively). The proportion of type I fibers did not reach statistically significant correlation with VO_2_max (Figure 1– C).

To evaluate the role of muscle function and morphology as independent determinants of VO_2_max, bivariate models were performed to include cardiac index as the variable representative of hemodynamic status (Table 5). In both bivariate models, the muscular variable analyzed was the result of an independent determination of VO_2_, showing that the overall exercise capacity, as measured by the VO_2_max, is directly influenced by peripheral muscular function and morphology and is independent of the hemodynamic status assessed through the cardiac output level.

**Table 5:** Bivariate linear regression models with VO_2_max (ml/kg/min) as a dependent variable.

	Model 1		Model 2	
	R^2^ = 0.658		R^2^ = 0.788	
	Beta	p	Beta	p
Cardiac Index - l/min/m2	0.630	0.006*	0.837	0.002*
Total work at 60°/s – J	0.496	0.024*		
Type I fibers area – µm^2^			0.682	0.008*

## Discussion

Our study has shown that skeletal muscle characteristics are determinants of maximum exercise capacity, assessed through the determination of VO_2_max independently of cardiac function, which suggests that peripheral muscular abnormalities are independent determinants of exercise intolerance in PAH.

Our study population was composed of idiopathic PAH and Sch-PAH patients, but their functional, clinical and hemodynamic impairments were not significantly different (data not shown). The clinical and hemodynamic profiles of our patients were compatible with previously described populations. PAH patients had a diminished exercise tolerance characterized by NYHA/WHO functional class II and decrements in PCS in SF-36 when compared with controls. Objective measurements of exercise performance (6MWD and VO_2_max) were consistent with these findings.

Although PAH patients presented lower lean total body mass, no significant difference in thigh muscle area was observed when measured by CT. Similar findings have previously described for muscle quantification through CT in PAH as well [8], but have been recently challenged by the study of Batt et al. that demonstrated muscle atrophy in patients with PAH [26]. The authors based their different findings on the hemodynamic severity of their patients. Our study was composed by PAH patients with hemodynamic severity similar to the one described by Batt et al. but muscle quantification through CT was not different from controls, which raises questions about the sensitivity of this method in detecting small differences. Differences in thigh muscle area may be more significant for patients with advanced muscle depletion, as is found in COPD patients [27]. The total body composition based on the impedance to electrical current flow had not been previously evaluated in PAH patients despite the low cost and feasibility of this technique. However, this approach has been extensively validated in patients with COPD and heart failure [28], [29], and our finding of lower lean mass in PAH is consistent with observations in other chronic conditions [30], [31].

We also demonstrated a significant reduction in respiratory and in peripheral musculature function. Significant reductions in MIP and MEP were found in PAH patients when compared with the control group, as previously described by Meyer et al. [9]. In heart failure, decreased MIP is associated with worse prognosis [32]; moreover, patients with decreased MIP who underwent muscular training had improved quality of life and functional capacity [33]. We found a significant correlation between MIP and VO_2_max and reduced performance in patients with inspiratory weakness. Quadriceps muscle function, in terms of force generated during short repetitions and in endurance, was also decreased in PAH patients as compared to controls. The association between respiratory and peripheral muscle impairment suggests a more skeletal muscular abnormality in PAH. Our study makes an innovative contribution to the literature by confirming this skeletal muscle involvement (respiratory and peripheral) in the same patient population and through comparisons with controls.

Exercise capacity was also reduced in PAH patients; both CPET and 6MWT measurements were decreased in the PAH group. Although PAH patients walked more than 450 meters in the 6MWT, which is considered to reflect a good prognosis [34], this distance was significantly less than the distance walked by the control group. In terms of maximal exercise evaluated by the CPET, PAH patients had significantly reduced VO_2_max, as has previously been described [35]. Our control group, composed by not physically active individuals without any cardiorespiratory symptom or limitation, presented a borderline VO_2_max level, which might be related to an early limitation due to limb fatigue on cycle, as suggested by the higher leg Borg index and a large chronotropic reserve. Altogether, these findings suggest some degree of deconditioning in this untrained group of health volunteers.

Most of the exercise limitation in PAH is attributed to reduced cardiac output or to a reduced cardiac reserve during exercise [36]. However, recent studies suggest the presence of morphological abnormalities in peripheral muscles that could also play a role in this limitation [8] [26]. Consistent with left heart failure, PAH patients also had an increased systemic inflammatory response [37], low cardiac output with impaired oxygen delivery [36] and sympathetic hyperactivity [38]. Accordingly, comparable alterations in muscle morphology and metabolism could also be found in PAH [26]. Our study supported this finding by confirming a reduction in type I fibers, as compared to previously published data from healthy subjects [25], suggesting a shift from an oxidative to a glycolytic pattern in the peripheral muscles of PAH patients. Although we have not performed a more direct measure of oxidative metabolism, a previous investigation found a reduced oxidative capacity in skeletal muscles in PAH [39]. Moreover, the observed reduction in the proportion on type I fibers has clinical implications, because it was associated with and was also an independent determinant of lower VO_2_max. However, the analysis of the proportion of type I fibers has to take into account its intrinsic limitations. The lower proportion of type one fibers could be representative of a PAH related myopathy but it could be solely a consequence of muscle atrophy related to a decondition/disuse. Our data do not allow the distinction between both patterns; nevertheless, our findings suggest that regardless of the primary cause of these skeletal muscle abnormalities, they have significant impact on the exercise limitation seen in PAH patients.

A significant correlation between VO_2_max and muscular functional variables was observed; nevertheless, the same phenomenon exists regarding VO_2_max and cardiac output. A borderline correlation between VO_2_max and the percentage of type I fibers in the muscle biopsy was also observed. To evaluate whether the association between VO_2_max and the muscular variables was independent of the hemodynamic profile, bivariate linear regression models showed that even when correcting for cardiac index, a significant association between maximal consumption and muscular function and structure still remains. A more comprehensive multivariate model could not be used because of the risk of overfitting, as one of the limitations of our study is the limited sample size of our study group. Even in light of this limitation, our study demonstrated that an altered muscular pattern is independently associated to exercise limitation in PAH and could possibly be used accordingly, as has been suggested in recently published rehabilitation studies [11], [12]. Although previous studies have addressed the involvement of peripheral muscles in PAH [8], [10], our results are the first to confirm the role of quadriceps weakness on decreased VO_2,_ even after adjusting for cardiac output in both the correlation analyses and the regression model.

The main limitation of the current study is the lack of parameters from muscle biopsies in controls but our intention was only to describe characteristics of fiber structures and distribution; the expected patterns in normal individuals are extensively defined and we used previous data obtained from a population with similar age [25]. Additionally, we have not performed a more comprehensive analysis of muscle characteristics such as metabolic enzymes, proteolysis and mitochondrial characteristics in PAH. Thus, we cannot rule out that the morphometric changes in the peripheral muscles are not related to a deconditioning component, which might also be present in these patients. Measurement of cardiac function at different levels of exercise would also have allowed a better understanding of the limitation imposed by cardiac output and the changing in its magnitude according to the effort level. Instead, we have used rest hemodynamics, thus limiting the extrapolation of our data to the whole spectrum of exercise activity.

In conclusion, our study has shown the coexistence of ventilatory and quadriceps weakness in the face of exercise intolerance in the same group of PAH patients. More interestingly, this study demonstrated an independent association between muscular pattern and maximum exercise capacity in PAH, independent of cardiac output. Future studies should evaluate whether this finding remains after specific muscular training and should also demonstrate the safety and efficacy of such training programs.
